# Differences in Intersubject Early Readiness Potentials Between Voluntary and Instructed Actions

**DOI:** 10.3389/fpsyg.2020.529821

**Published:** 2020-09-28

**Authors:** Lipeng Zhang, Rui Zhang, Dezhong Yao, Li Shi, Jinfeng Gao, Yuxia Hu

**Affiliations:** ^1^School of Electrical Engineering, Zhengzhou University, Zhengzhou, China; ^2^Henan Key Laboratory of Brain Science and Brain–Computer Interface Technology, Zhengzhou, China; ^3^Key Laboratory for Neuroinformation, University of Electronic Science and Technology, Chengdu, China; ^4^Department of Automation, Tsinghua University, Beijing, China; ^5^Beijing National Research Center for Information Science and Technology, Beijing, China

**Keywords:** EEG, readiness potential, voluntary action, instructed action, slow cortical potential

## Abstract

Readiness potential (RP) is a slow negative electroencephalogram (EEG) potential prior to voluntary action and was first described by Kornhuber and Deecke (1965). Recent studies have demonstrated that a few subjects do not exhibit standard RP before voluntary action. In our previous study, we also found that some subjects did not show an early RP preceding instructed action. Although this phenomenon may be meaningful, no studies have yet investigated its origins. In the present study, we designed and implemented an experimental paradigm involving voluntary and instructed actions in the form of hand movements from 29 subjects with concurrent acquisition of EEGs. According to whether the subjects showed a standard RP waveform during instructed action, they were divided into the SHOW and NOSHOW group. Then, the RPs and voltage topographies were plotted for each group. Finally, the slope of each epoch at the early RP phase was estimated. We showed that early RPs were absent in 14 of 29 subjects during instructed actions. Besides, based on the slow cortical potential (SCP) sampling hypothesis, we also showed a decreased proportion in the negative potential for the NOSHOW group. Our results suggested that early RP is absent among approximately half of subjects during instructed action and that the decreased proportion of negative potential shifts may account for the absence of early RP in the NOSHOW group.

## Introduction

Readiness potential (RP) is a slow-rising negative electrocortical activity initiated approximately 2 s before voluntary action. Kornhuber and Deecke (1965) first discovered the RP in 1965 but were unable to elucidate its neural mechanism. Conventionally, the RP is interpreted as an indicator of motor preparation and appears only before voluntary action (Libet, 1985; Passingham, 1993; Ball et al., 1999; Cunnington et al., 2002; Shibasaki and Hallett, 2006). The RP prior to voluntary actions is not easily detected on individual trials due to its small amplitude compared to that of background neural activity in electroencephalogram (EEG) recordings (Shibasaki and Hallett, 2006; Khalighinejad et al., 2018; Schurger, 2018; Travers et al., 2019). The RP is typically obtained by averaging a large number of trials (Travers et al., 2019) because it is time-locked to the beginning of the action. The RP is usually divided into two components, namely, early and late RPs (Di Russo et al., 2017). The generation of the early RP can be tracked to the supplementary motor area (SMA) as well as the pre-SMA, while the late RP mainly occurs in contralateral motor and premotor cortices (Deecke et al., 1976; Shibasaki and Hallett, 2006); thus, the early and late RPs are considered to reflect different motor preparatory processes.

Based on the generative process of the RP, a stochastic decision model was proposed by Schurger et al. (2012) and Schurger (2018), which assumes that the RP may reflect subthreshold stochastic fluctuations in neural activity. Schmidt et al. proposed a different hypothesis that is called the selective slow cortical potential (SCP) sampling hypothesis (Jo et al., 2013; Schmidt et al., 2016), which posits that the initiation of a voluntary action is more likely to occur during negative fluctuations of the SCP and that the sampling and averaging of many trials lead to the observed negative potential. Although the RP has been extensively studied, a consensus on its neural basis has still not been reached.

An instructed action involves directly responding to an external cue, such as a response to a verbal command (Passingham et al., 2010; Khalighinejad et al., 2019). In contrast, a voluntary action consists of an endogenous, self-initiated action that requires no external cue (Fried et al., 2017). In general, the RP has been associated with voluntary action, but several studies have also reported the presence of a similar slow-rising negative potential for instructed action (Jenkins et al., 2000; Di Russo et al., 2005; Berchicci et al., 2016). Furthermore, several neuroimaging studies have found similar brain activations during voluntary and instructed actions (Di Russo et al., 2016, 2017). In our previous study, to avoid the influence of induced neural activity by the experimental cue, subjects were required to move their hand at 2 s after the cue disappeared (Hu et al., 2017). Interestingly, under such conditions, we found that the early RP was absent in some subjects during instructed action. Corroborating our previous findings, some other studies have also reported that some subjects do not exhibit an early RP during instructed action (Schurger et al., 2012; Parés-Pujolràs et al., 2019). In most cases, these subjects were excluded from further analysis.

In the present study, in order to investigate differences in RPs between subjects, we designed an experimental paradigm in which subjects performed both voluntary and instructed actions during concurrent EEG recordings. Thereafter, these subjects were divided into two groups according to whether the standard RP waveform was present during instructed action. Then, RPs and scalp voltage topographies were plotted for analysis. Finally, based on the SCP sampling hypothesis, the slope of each epoch at the early RP phase was estimated.

## Materials and Methods

### Subjects

Twenty-nine healthy subjects (S1–S29, 27 ± 1.8 years old, nine female, all right-handed) recruited from Zhengzhou University participated in the present study. All subjects had normal or corrected-to-normal vision, reported having normal hearing, and had no history of neurological or psychiatric illness. Prior to the experiment, each subject was informed of the experimental procedure and signed a letter of consent. The present study followed the principles of the Declaration of Helsinki.

### Experiments

#### Experiment 1: Instructed Action

**FIGURE 1:**
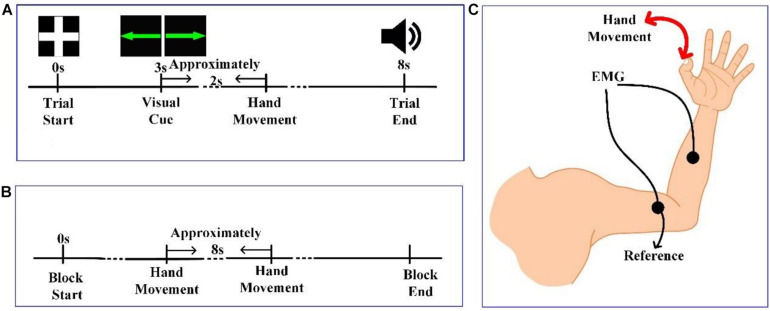
**(A)** The timeline of a trial in experiment 1. **(B)** The timeline of a session in experiment 2. **(C)** Diagram of hand movement and positioning of electromyographic (EMG) electrodes.

At the beginning of each trial, a white cross was presented in the center of the screen, as shown in Figure 1A. For the next 3 s, subjects remained still, with their hands, forearms, and elbows resting on the armrests of the chair. Next, a green line with an arrow pointing either left or right appeared in the center of the screen for 0.5 s. After the cue disappeared, the subjects prepared to perform the corresponding task instructed by the visual cue (left-hand movement for left-pointing arrow, LH; right-hand movement for right-pointing arrow, RH). After a delayed time of approximately 2 s, the subjects performed the corresponding hand movement. Then, 5 s after the visual cue, an auditory cue was presented to inform the subject that the current trial was over. The LH and RH tasks were presented randomly. The time interval between consecutive trials was 2.5–3.5 s. Ten sessions were conducted for each subject, with 30 trials per session—15 trials each for the LH task and RH task.

#### Experiment 2: Voluntary Action

In experiment 2, there was no visual cue, and subjects performed LH and RH tasks voluntarily. At the beginning of each session in Figure 1B, the experimenter reminded the subject to stay still and that the experiment would begin shortly. Then, subjects were allowed to perform hand movements at any time that they wanted. However, we instructed subjects to keep the time interval between adjacent trials to approximately 8 s. Each session lasted 5 min, and 10 sessions were conducted for each subject.

#### Experimental Environment

Each subject was seated in a comfortable chair in a room with normal lighting and temperature. The subject faced a screen and was asked to focus on the central point of the screen. In experiment 2, the screen was closed, and a fixation point remained in the middle of the central point. The distance between the subject and the screen was 80 cm, and the screen’s central point and the subject’s eyes were at the same height. During the EEG recording, the subject was asked to avoid eye movements, swallowing, and unnecessary limb movements. All 29 subjects completed both experiments 1 and 2, and the order in which the experiments were completed for each subject was random.

#### Experimental Setup

**FIGURE 2:**
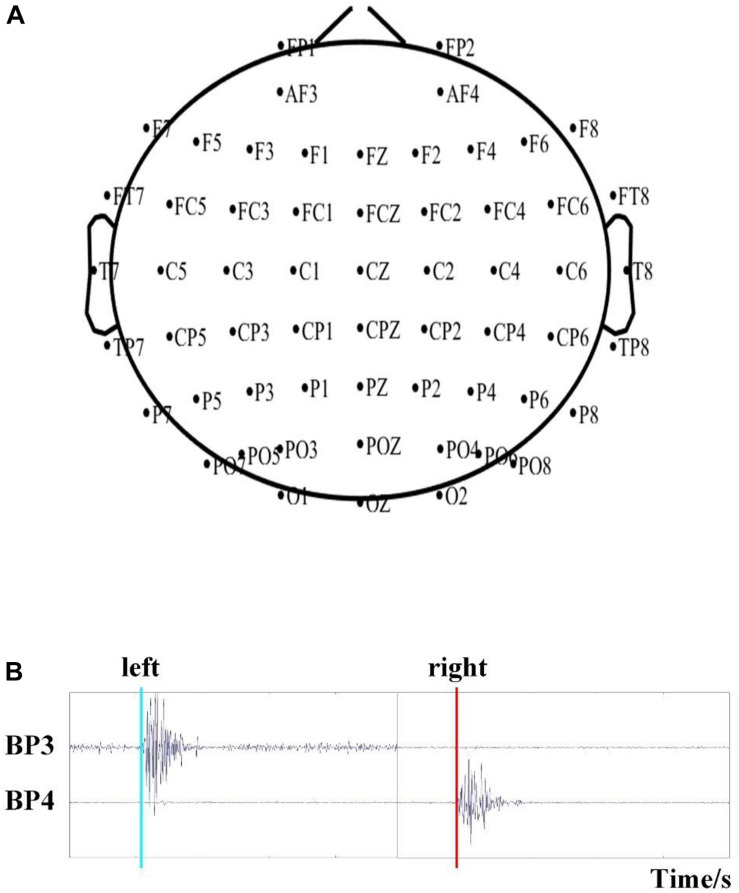
**(A)** The position of the selected 59 EEG electrodes. **(B)** Detection of the time of movement onset based on EMG recordings.

Brain activity was recorded using a Neuroscan NuAmps digital amplifier system with 64 electrodes. DC-EEG recordings were arranged in the standard 10–20 EEG configuration. Among the 64 electrodes, 59 EEG electrodes were selected, and their positions are shown in Figure 2A. All superficially located brain regions were covered by these electrodes. Horizontal and vertical electrooculograms (HEOGs and VEOGs) were additionally recorded with bipolar montages using electrodes at left and right external canthi for HEOGs and below and above the left eye for VEOGs. Compared with recording action onset by pressing a key, obtaining action onsets via EMG is more advantageous. Two extended bipolar channels (BP3 and BP4), which consisted of two electrodes (one of which was the reference electrode, Figure 1C), were used to acquire the EMG data from the left and right arms of each subject. The EEG data were acquired at a sampling rate of 250 Hz with the Cz reference (Cz-REF) as a reference, and the impedance of all electrodes was less than 5 kΩ. The Cz-REF was located between the Cz and CPZ electrodes. The stimulus program was written via the E-Prime software.

### Data Processing

#### Subject Groupings

To evaluate the RP’s waveform, we assumed that the RP’s waveform of each subject during voluntary action was standard. The RP’s correlation coefficients between voluntary and instructed actions were calculated for each subject. If the correlation coefficient was lower than 0.6, the subject would be included in the NOSHOW group. So subjects were divided into two groups: the SHOW (15 subjects) group and the NOSHOW (14 subjects) group. Hence, our grouping resulted in eight conditions: 2 experiments (instructed/voluntary) × 2 groups (SHOW/NOSHOW) × 2 hands (LH/RH).

#### EMG Analysis

The onsets of LH/RH actions were determined via EMG recordings, as previously described (Hu et al., 2017). The EMG data were filtered by a basic finite-impulse response filter with cutoff frequencies of 6 and 50 Hz. We then calculated the energy of the filtered data and set a proper threshold to detect the onset time of action, as shown in Figure 2B. We recorded the onset times in a TXT file for further analysis.

#### Behavioral Analysis

In order to confirm whether there was a significant difference in behavior between the two groups of subjects during instructed action, the individual latency from the cue onset to EMG onset for each trial in instructed action experiments was calculated. Additionally, the difference between the LH and RH during the instructed action was also calculated.

#### EEG Preprocessing

Eye movement artifacts were removed by Scan 4.5 software, and the threshold was set at 60 μV. The processed data were then imported to the EEGLAB toolbox (Delorme and Makeig, 2004) for further analysis.

All channels were 0.1–30-Hz band-pass filtered with a zero-phase shift filter. According to our previous results, the reference electrode standardization technique (REST) reference is appropriate for RP analysis (Hu et al., 2017). Hence, the EEG signals were re-referenced to the REST reference (Yao, 2001, 2017; Yao et al., 2007; Dong et al., 2017). To remove other artifacts, for example, EMG and ECG data, independent component analysis (ICA) was performed (Jung et al., 2000). All rejected artifacts were removed manually.

#### EEG Analysis

The preprocessed EEG signals were segmented in epochs starting at 2.1 s prior to the EMG onset (time 0) and lasting for 4.1 s, with the baseline corrected based on the initial 0.1 s (−2.1 to −2.0 s). Epochs with an absolute EEG signal amplitude over 80 μV were considered to be contaminated by noise and were discarded before further analysis; on average, approximately 20% of the epochs were discarded. The remaining epochs were averaged based on different conditions. For each condition, grand-averaged RPs and scalp topographies were plotted. The average amplitudes of the early and late RPs at the FCz site were calculated for statistical analysis.

According to the SCP sampling hypothesis, a voluntary movement is initiated more often during an ongoing negative cortical potential than during a positive potential (Schmidt et al., 2016). During instructed action, we assumed that the cue would modulate the time of initiation and may be related to the absence of an early RP. Hence, we estimated the slope of each epoch at the early RP phase as follows.

The segmented EEG ranging from 2.1 to 0.5 s before the EMG onset was applied for further analysis. To reduce noise, we averaged the 15 electrodes nearest to the FCZ electrode (FZ, F1, F2, F3, F4, FCZ, FC1, FC2, FC3, FC4, CZ, C1, C2, C3, and C4). The averaged signals were segmented into 16 non-overlapping segments and were averaged for each segment. Then, the slope of each epoch was estimated by fitting a first-order polynomial function (Jo et al., 2013). According to either a negative or positive slope, each epoch was classified as either a negative (*k* < 0) or positive (*k* > 0) epoch (if *k* = 0, this trial was rejected). Subsequently, both negative and positive epochs were averaged separately for voluntary action and instructed action in the SHOW and NOSHOW groups.

### Statistical Analysis

In the current study, two-sample *t*-tests were utilized to quantify differences between the SHOW and NOSHOW groups. For the LH and RH, paired *t*-tests were used. All statistics were carried out using MATLAB 2016b software.

## Results

### Behavioral Data

**FIGURE 3:**
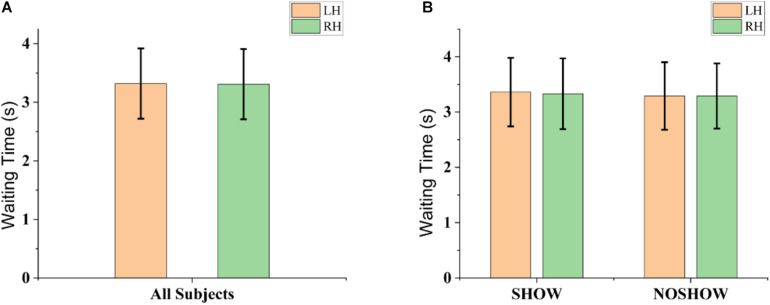
Behavioral indicators of performance during instructed action. **(A)** Grand-averaged latency from the cue onset to EMG onset for the LH and RH. **(B)** Grand-averaged latency from the cue onset to EMG onset for the SHOW and NOSHOW groups for the LH and RH. Data are reported as the mean ± SD.

Behavioral indicators of performance during instructed action are shown in Figure 3. As shown in Figure 3A, there was no significant difference between the LH and RH in terms of the latency from the cue onset to EMG onset (LH: 3.32 ± 0.60 s; RH: 3.31 ± 0.60 s; *p* > 0.05). As shown in Figure 3B, the latency also did not show significant difference in the SHOW (LH: 3.36 ± 0.62 s; RH: 3.33 ± 0.64 s; *p* > 0.05) and NOSHOW groups (LH: 3.29 ± 0.61 s; RH: 3.29 ± 0.59 s; *p* > 0.05) between the LH and RH. Importantly, there was no significant difference between the SHOW group and NOSHOW group (SHOW and LH vs. NOSHOW and LH: *p* > 0.05; SHOW and RH vs. NOSHOW and RH: *p* > 0.05) for the LH or RH. Hence, no significant difference was found in terms of the behavior results for the LH/RH comparison or SHOW/NOSHOW group comparison.

### RP and Scalp Topography in the SHOW Group

**FIGURE 4:**
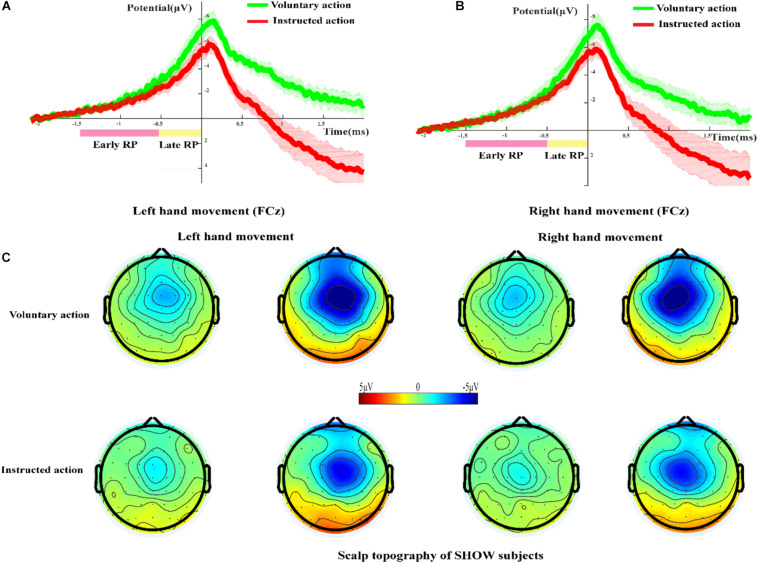
The RP and scalp topography in the SHOW group. **(A,B)** Grand-averaged RP waveforms during voluntary action and instructed action in the SHOW group at the FCz site (**A**, left-hand movement; **B**, right-hand movement). **(C)** Scalp topography of the early and late RPs during voluntary action and instructed action in the SHOW group.

Figure 4A shows the grand-averaged RP waveform during voluntary action and instructed action for the SHOW group at the FCz site. The RP originated at approximately 2 s before EMG onset and reached its maximum value at about 0.1 s. As shown in Figure 4A, for the LH condition, the early RP waveforms between voluntary and instructed actions were nearly the same. Compared with that during instructed action, the late RP during voluntary action had a higher amplitude, but statistical analysis revealed that there was no significant difference in terms of this amplitude (*p* > 0.05). Figure 4B shows similar results for the RH condition.

Figure 4C shows the scalp topography of the early and late RPs during voluntary action and instructed action in the SHOW group. The frontal areas were obviously activated during voluntary and instructed conditions, and there was significant lateralization in the late RP phase. Additionally, the activated brain regions during voluntary action were larger than those during instructed action.

### RP and Scalp Topography in the NOSHOW Group

**FIGURE 5:**
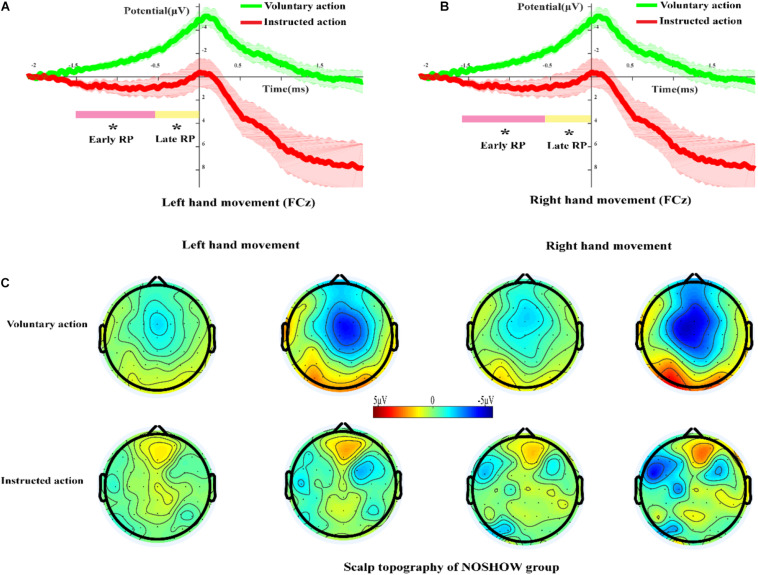
The RP and scalp topography in the NOSHOW group. **(A,B)** Grand-averaged RP waveform of the voluntary/instructed actions for the NOSHOW group at the FCz site (**A**, left-hand movement; **B**, right-hand movement). **(C)** Scalp topography of the early and late RPs for voluntary/instructed actions in the NOSHOW group (*p < 0.05).

Figure 5A shows the grand-averaged RP waveforms of the LH voluntary and instructed actions for the NOSHOW group at the FCz site. At the early and late RP phases, there was a standard RP during voluntary action. However, during instructed action, an early RP was absent, and the corresponding waveforms approximated a horizontal line. Until the late RP phase, a slowly rising negative wave appeared. In terms of the average amplitude of the RP, there was significant difference for the LH in the early RP (*p* < 0.05) and the late RP (*p* < 0.05) between voluntary and instructed actions. Similar results were achieved for the RH (Figure 5B) in that there was a significant difference in the early RP (*p* < 0.05) and late RP (*p* < 0.05).

Figure 5C shows the scalp topography of the early and late RPs for voluntary and instructed actions during the NOSHOW group. For the voluntary action, as expected, the SMA was activated during the early RP. The M1 area and SMA were activated in the late RP phase. For LH/RH movement, the brain activation regions were lateralized during the late RP phase. However, for the instructed action, we were unable to clearly discern differential areas of activation during the early and late RP phases.

### Differences in the Early RP Slope Between the SHOW and NOSHOW Groups

**FIGURE 6:**
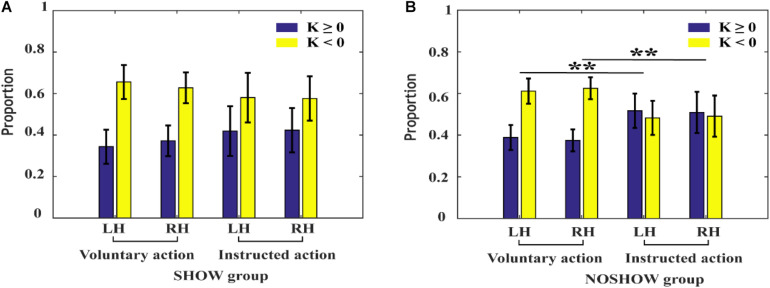
The proportion of negative and positive shifts during voluntary/instructed actions. **(A)** SHOW group, **(B)** NOSHOW group (**p < 0.01).

The proportions of negative and positive potential shifts during voluntary/instructed actions are shown in Figure 6. For the SHOW group, there was no significant difference in the proportions of negative and positive potential shifts between voluntary and instructed actions (approximately 0.7:0.3). However, for the NOSHOW group, compared with that during voluntary action, the proportion of negative shifts was significantly decreased during instructed action (LH: *p* < 0.01; RH: *p* < 0.01).

## Discussion

The present study investigated intersubject differences in the presence/absence of the early RP during voluntary and instructed actions. We found that the early RP was absent in 14 of 29 subjects while performing instructed action. Based on the RP waveforms, subjects were first divided into SHOW and NOSHOW groups. The behavioral results during instructed action were analyzed and revealed that there was no significant behavioral difference between the SHOW and NOSHOW groups. Then, the RPs and scalp topographies were plotted for the two groups. For the NOSHOW group, we found that the early RP was absent and that the late RP had a smaller amplitude compared to that of the SHOW group. Finally, the proportions of negative and positive potential shifts for the two groups were calculated and revealed that negative potential shifts were significantly decreased during instructed action compared with those during voluntary action in the NOSHOW group.

Previous studies have also reported an absence of the RP in some subjects during voluntary action (Schurger et al., 2012; Parés-Pujolràs et al., 2019), but few such subjects were found, and these subjects were often excluded from further analysis. In our present study, all subjects showed an RP during voluntary action. However, during instructed action, an early RP was absent in 9 of the 17 subjects. Due to the different experimental tasks (voluntary/instructed) and the relatively large number of NOSHOW subjects, we hypothesize that these NOSHOW RP phenomena occurred for different reasons. The former situation may have been due to either poor-quality EEG signals or subject-specific reasons. In terms of our present results, previous studies have reported that both motor timing and time estimation tasks are associated with activation in some of the same regions that are associated with voluntary action, most notably the SMA and dorsolateral prefrontal cortex (dlPFC) (Rubia and Smith, 2004).

Unlike the early RP, although the late RP was present during instructed action for both the SHOW and NOSHOW groups, its amplitude was small compared with that during voluntary action. These results confirm that the early and late RPs signify different cognitive processes (Shibasaki and Hallett, 2006) and are modulated by different factors. There are two pieces of evidence to support this conclusion. First, Shibasaki and colleagues reported that the early RP originated in the SMA, while the late RP occurred in the contralateral motor and premotor areas (Shibasaki and Hallett, 2006). Second, Eimer and colleagues confirmed that the RP begins symmetrically; however, before movement, the RP lateralizes, with stronger amplitudes observed over the hemisphere contralateral to the effector performing the movement (Eimer, 1998). Later studies also confirmed these results from different technical perspectives (Haggard, 2008; Fried et al., 2011; Nguyen et al., 2014; Khalighinejad et al., 2018). Hence, our results are consistent with the conclusions of previous studies. Additionally, the main reason for the smaller late RP amplitude may be that the amplitude does not increase cumulatively during the early RP stage.

Based on the SCP sampling hypothesis (Schmidt et al., 2016), we hypothesize that the cue during instructed action can modulate the timing of action preparation and the proportion of negative and positive potential shifts. Our present results are consistent with this hypothesis. For the NOSHOW group, the proportion of negative to positive potential shifts is close to 0.5:0.5 during instructed action; compared to that during voluntary action (about 0.6:0.4), this difference was statistically significant. These results indicate that the decreased proportion of negative potential shifts may account for the absence of the early RP in the NOSHOW group.

According to the above results, we assume that intersubject differences in the presence/absence of the early RP during instructed action may be due to different strategies during task execution. We then investigated the execution strategies chosen by the participants when performing the instructed action. Since the subjects were asked to wait for approximately 2 s and then perform the hand movement task after the cue appeared, the following two strategies were adopted: (1) subjects focused on the control of preparation time and (2) subjects focused on the control of movement preparation. Our results showed that seven subjects in the SHOW group chose the second of these strategies, whereas all of subjects in the NOSHOW group chose the first of these strategies to complete the tasks. However, the evidence provided by our *post-hoc* analysis was not strong enough. Hence, it remains unclear whether focusing on preparation time is the main reason for the disappearance of early RP and still requires more rigorous controlled experiments to verify.

## Limitation

The grouping criteria between NOSHOW and SHOW groups in the study were not used during the previous researches. So more pieces of evidence are needed for the grouping criteria in the future.

## Data Availability Statement

The datasets generated for this study are available on request to the corresponding author.

## Ethics Statement

The studies involving human participants were reviewed and approved by the ethics committee of the Zhengzhou University. The patients/participants provided their written informed consent to participate in this study.

## Author Contributions

LZ: conceptualization, methodology, software, and writing–original draft preparation. RZ: conceptualization, methodology, software, and writing–reviewing and editing. DY: conceptualization, methodology, and data curation. LS: investigation, funding acquisition, and supervision. JG: investigation, supervision, and validation. YH: conceptualization, project administration, funding acquisition, investigation, and supervision. All authors contributed to the article and approved the submitted version.

## Conflict of Interest

The authors declare that the research was conducted in the absence of any commercial or financial relationships that could be construed as a potential conflict of interest.
